# Global academic response to COVID‐19: Cross‐sectional study

**DOI:** 10.1002/leap.1317

**Published:** 2020-07-01

**Authors:** Jack A. Helliwell, William S. Bolton, Joshua R. Burke, Jim P. Tiernan, David G. Jayne, Stephen J. Chapman

**Affiliations:** ^1^ Leeds Institute of Medical Research at St. James's University of Leeds Leeds LS9 7TF UK

**Keywords:** coronavirus, SARS‐CoV‐2, dissemination, open‐access, data sharing

## Abstract

This study explores the response to COVID‐19 from investigators, editors, and publishers and seeks to define challenges during the early stages of the pandemic. A cross‐sectional bibliometric review of COVID‐19 literature was undertaken between 1 November 2019 and 24 March 2020, along with a comparative review of Middle East respiratory syndrome (MERS) literature. Investigator responsiveness was assessed by measuring the volume and type of research published. Editorial responsiveness was assessed by measuring the submission‐to‐acceptance time and availability of original data. Publisher‐responsiveness was assessed by measuring the acceptance‐to‐publication time and the provision of open access. Three hundred and ninety‐eight of 2,835 COVID‐19 and 55 of 1,513 MERS search results were eligible. Most COVID‐19 studies were clinical reports (*n* = 242; 60.8%). The submission‐to‐acceptance [median: 5 days (IQR: 3–11) versus 71.5 days (38–106); *P* < .001] and acceptance‐to‐publication [median: 5 days (IQR: 2–8) versus 22.5 days (4–48·5‐; *P* < .001] times were strikingly shorter for COVID‐19. Almost all COVID‐19 (*n* = 396; 99.5%) and MERS (*n* = 55; 100%) studies were open‐access. Data sharing was infrequent, with original data available for 104 (26.1%) COVID‐19 and 10 (18.2%) MERS studies (*P* = .203). The early academic response was characterized by investigators aiming to define the disease. Studies were made rapidly and openly available. Only one‐in‐four were published alongside original data, which is a key target for improvement.

**Key points:**

- COVID‐19 publications show rapid response from investigators, specifically aiming to define the disease.
- Median time between submission and acceptance of COVID‐19 articles is 5 days demonstrating rapid decision‐making compared with the median of 71.5 days for MERS articles.
- Median time from acceptance to publication of COVID‐19 articles is 5 days, confirming the ability to introduce rapid increases at times of crisis, such as during the SARS outbreak.
- The majority of both COVID‐19 and MERS articles are available open‐access.

## INTRODUCTION

Coronavirus disease 2019 (COVID‐19) caused by the severe acute respiratory syndrome coronavirus 2 (SARS‐CoV‐2) has spread rapidly since it began in the city of Wuhan in late 2019 (Xu *et al*., 2020). The outbreak has been declared as a pandemic by the World Health Organization (WHO), reflecting its spread to more than 200 countries worldwide (WHO, 2020c). As of April 2020, there have been more than 100,000 deaths and the number of those affected continues to rise (WHO, 2020a).

In response to this unprecedented situation, healthcare systems around the world have taken urgent action to scale up medical staffing, equipment, and infrastructure. In the UK, this is demonstrated by the construction of new field hospitals, the return of thousands of retired healthcare workers, and the re‐purposing of many acute and urgent hospital services (NHS England, 2020). The role that science and data play in the face of the COVID‐19 pandemic must also be recognized. Robust and rapidly available evidence is essential for improving the detection and treatment of the virus as well as for reducing its transmission. It is the responsibility of all members of the academic community to facilitate this transfer of knowledge so that patients around the world receive the best evidence‐based care available.

There is limited information about how the academic community has responded to COVID‐19 and how this can be further guided during times of global crisis. There is a need for investigators to respond urgently to new clinical priorities across all sectors of healthcare and to work in unfamiliar environments. Journal editors must facilitate rapid and robust peer review while making difficult editorial decisions based on little previous academic background. Publishers must mobilize quickly to enable timely publication of manuscripts so that research outputs can be realized and implemented into practice. An early understanding of this response is important so that the academic community can identify early challenges and respond appropriately as the COVID‐19 pandemic develops.

## MATERIALS AND METHODS

### Ethics and governance

As a review of published literature, approval by a research ethics committee was not applicable. Since only bibliometric outcomes were considered, rather than outcomes of direct relevance to research participants, this review was not eligible for registration on the PROSPERO database. There were no changes to the research design or outcomes during the course of the study. The results are reported with consideration to the Preferred Reporting Items for Systematic Reviews and Meta‐Analyses (PRISMA) Checklist (Liberati *et al*., 2009).

### Aims and objectives

The study aimed to examine the responsiveness of the academic community to COVID‐19 during its early stages. The following objectives were predefined:

- To explore investigator responsiveness by describing the volume and type of research accepted in peer‐reviewed journals.
- To explore editorial responsiveness by describing the time taken to facilitate editorial/peer review and the availability of original data as a condition of publication.
- To explore publisher responsiveness by describing the time taken for accepted manuscripts to be available and the provision of open‐access publication.

### Study design

This was a systematic, cross‐sectional, bibliometric review of existing healthcare literature related to COVID‐19. To facilitate comparisons of outcomes in a unique setting, a comparable review of Middle East respiratory syndrome (MERS) was also performed and used as control. MERS was chosen as a suitable comparator because it similarly represents a respiratory illness caused by a zoonotic coronavirus, but was not classified as a pandemic or a Public Health Emergency of International Concern by the WHO during the search period (WHO, 2020d). It therefore represents a non‐COVID‐19 and non‐pandemic control.

### Definitions

In line with the WHO, COVID‐19 describes the disease caused by the severe acute respiratory syndrome coronavirus 2 (SARS‐CoV‐2) and MERS describes the disease caused by the Middle East respiratory syndrome‐related coronavirus (MERS‐CoV) (WHO, 2020b). The early stages of COVID‐19 was defined as the period between the first known human case and its classification as a pandemic on 11 March 2020 (WHO, 2020c). Availability of original data was considered to be an editorial responsibility since this is recommended by the International Committee of Medical Journal Editors for clinical trials and can be enforced as a condition of publication (Taichman *et al*., 2017). Open‐access was defined by the model of Gold open‐access, where articles and related content are freely accessible at the point of publication. Fully open‐access journals were defined by their listing on the Directory of Open Access Journals (https://doaj.org).

### Search strategy

Systematic searches of MEDLINE (via OvidSP) and EMBASE (via OvidSP) were performed by a single investigator on 25 March 2020. For COVID‐19, time limits were set between 1 November 2019 and 24 March 2020. This start date was chosen to represent the earliest plausible time of the first human report. The end date reflected the official WHO classification of COVID‐19 as a global pandemic (11 March 2020) along with a 14‐day lag period for manuscripts already in production. For MERS, a comparable search was performed between 1 November 2018 and 24 March 2019. The difference in year was chosen to represent a non‐COVID‐19 control period. The results of both searches were first de‐duplicated and saved offline. Two independent investigators then screened titles, abstracts, and full texts for possible inclusion, with discrepancies addressed through re‐examination and discussion. A full outline of search strategies is available in Table 1.

**Table 1:** COVID 19 and MERS search strategies (via OvidSP) (undertaken 25 March 2020)

COVID‐19	
1	SARS‐CoV‐2
2	nCoV‐19
3	2019‐nCoV
4	COVID‐19
5	Novel coronavirus
6	Severe acute respiratory syndrome coronavirus 2
7	1 OR 2 OR 3 OR 4 OR 5 OR 6
8	(Remove duplicates)
9	(Time limit: 2019–current)
10	(Limit to English)
MERS	
1	MERS
2	MERS‐CoV
3	Middle East respiratory syndrome
4	1 OR 2 OR 3
5	(Time limit: 2018–2019)
6	(Limit to English)
7	(Remove duplicates)

### Study eligibility criteria

For published studies, any COVID‐19‐ or MERS‐related manuscript available online or in print within the defined time periods was eligible for inclusion. Eligible studies had to report primary data. This included studies that performed analyses using open source data sets, but not studies that reported systematic reviews or meta‐analyses. Studies of animals or pre‐clinical models were eligible, but only if the outcomes contributed to human rather than veterinary medicine. Articles published in non‐English languages and other grey literature (such as conference abstracts) were excluded since these are not practically accessible by the global academic community.

### Study outcomes

Academic responsiveness was considered across three groups of the academic community. Investigator responsiveness was explored by measuring the volume and type of research (pre‐clinical, clinical, and modelling) published in peer‐reviewed journals. Editorial responsiveness was explored by measuring the time taken for editorial/peer review, defined as the number of days between manuscript submission and acceptance. The availability of original data to support the results was also explored. Data were considered available if individual observations (or source code in the case of modelling studies) were provided as a supplementary file, in a controlled‐access repository, or if an explicit statement of availability from authors was present. Where the status of data sharing was unclear, corresponding authors were contacted and non‐responses were considered to represent an absence of data. Publisher responsiveness was explored by measuring the time taken for manuscripts to complete production, defined as the number of days between acceptance and first availability online or in print. Publisher responsiveness was also assessed by exploring the incidence of open‐access publication.

### Data collection

Data were collected by a single investigator and checked by an independent investigator for accuracy. Data sources included full‐text manuscripts and editorial‐related content. Data points of interest were country of origin (according to the corresponding institution), manuscript format (short communication/letter or full‐text), journal subject category, and subject category ranking (according to Thomas Reuters Journal Citation Reports). For clinical studies, additional data of interest were population (adults, children, pregnant adults, or healthcare workers), study design (case report, case series, observational, or interventional) and clinical focus (definition of disease, diagnosis/screening, treatment, prevention, or resource use). Definition of disease related to studies describing clinical features and outcomes.

### Statistical analysis

Data are presented descriptively using averages and measures of variance. Continuous data were analysed using the student's *t*‐test or analysis of variance (ANOVA). Categorical data were analysed using *χ* ^2^ or Fisher's exact test. All statistical comparisons of COVID‐19 and MERS studies were pre‐planned. A single sub‐analysis of data sharing practices was performed with the exclusion of case reports since these may or may not provide the full complement of data within the reported manuscript. The level of statistical significance was set at *P* < .05.

### Assessment of quality

Since wide heterogeneity in study design was expected, a systematic assessment of study quality was not feasible. Instead, compliance to reporting guidelines, as recommended by the Equator Network, was used as a broad, surrogate marker of manuscript reporting quality. Author declarations of compliance to the STROBE and CONSORT checklists (or other relevant checklists/extensions) were evaluated for all full‐text observational and interventional studies (Schulz, Altman, & Moher, 2010; von Elm *et al*., 2014).

## RESULTS

### Study characteristics

**Figure 1:**
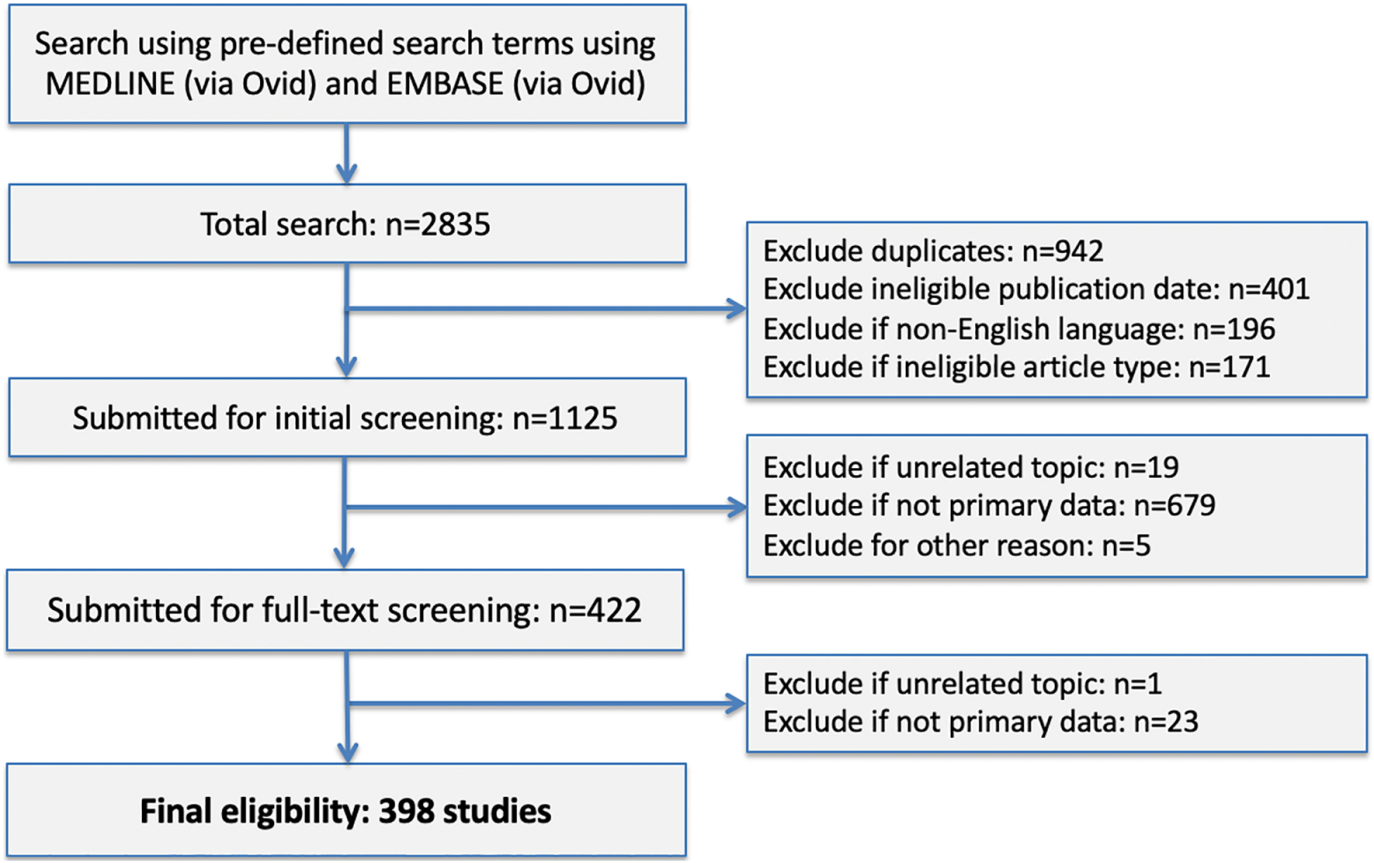
Flow diagram showing selection of eligible articles relating to COVID‐19.

**Figure 2:**
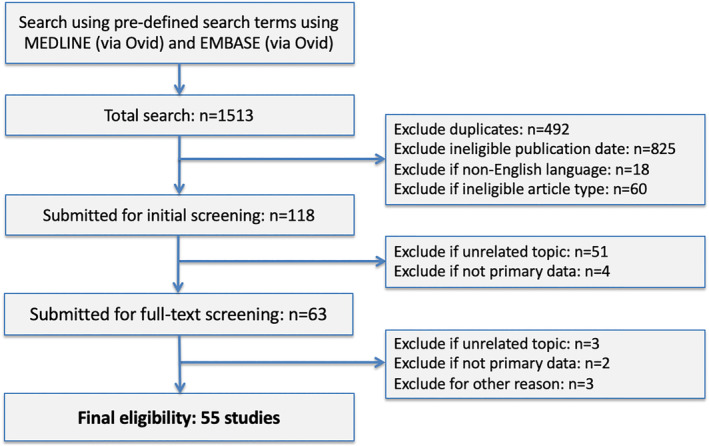
Flow diagram showing selection of eligible articles relating to MERS.

Across both searches, 398 of 2,835 COVID‐19 search results and 55 of 1,513 MERS search results were eligible for inclusion (Figs. 1 and 2). Lists of all included studies are available in Appendices S1 and S2 (Supporting Information). The majority of COVID‐19 studies were authored from institutions in China (*n* = 254; 63.8%) whereas the majority of MERS studies were authored from the USA (*n* = 14; 25.5%). A greater proportion of COVID‐19 studies were published as letters or short communications (*n* = 158; 39.7%) compared with MERS (*n* = 4; 7.3%). Infectious Diseases (*n* = 117; 29.4%) and General and Internal Medicine (*n* = 78; 19.6%) were the most common journal subject categories for COVID‐19, with the majority (*n* = 248; 62.3%) of manuscripts published in the first quartile of category‐specific rankings. In contrast, the most common category for MERS studies was Virology (*n* = 15; 27.3%) (Table 2).

**Table 2:** Bibliometric characteristics of COVID‐19 and MERS published studies

		COVID‐19 (*n* = 398)	MERS (*n* = 55)
Country of publication^a^	China	254 (63.8%)	7 (12.7%)
	USA	28 (7.0%)	14 (25.5%)
	Japan	13 (3.3%)	2 (3.6%)
	Korea	11 (2.8%)	12 (21.8%)
	Singapore	10 (2.5%)	0 (0.0%)
	Canada	9 (2.3%)	1 (1.8%)
	Italy	9 (2.3%)	0 (0.0%)
	Germany	8 (2.0%)	4 (7.3%)
	UK	8 (2.0%)	0 (0.0%)
	Saudi Arabia	1 (0.3%)	8 (12.7%)
	Others^b^	47 (11.8%)	7 (14.5%)
Format of publication	Letter/communication	158 (39.7%)	4 (7.3%)
	Full‐text manuscript	240 (60.3%)	51 (92.7%)
Type of research	Pre‐clinical	90 (22.6%)	38 (69.1%)
	Clinical	242 (60.8%)	13 (23.6%)
	Modelling	62 (15.6%)	1 (1.8%)
	Other/miscellaneous^c^	4 (1.0%)	3 (5.5%)
Journal category^d^	Infectious diseases	117 (29.4%)	10 (18.2%)
	Medicine, general and Internal	78 (19.6%)	1 (1.8%)
	Radiology, nuclear medicine, and medical imaging	44 (11.1%)	0 (0.0%)
	Virology	39 (9.8%)	15 (27.3%)
	Microbiology	16 (4.0%)	6 (10.9%)
	Multidisciplinary sciences	8 (2.0%)	3 (5.5%)
	Others^e^	96 (24.1%)	20 (36.4%)
Journal category quartile^f^	First	248 (62.3%)	20 (36.4%)
	Second	50 (12.6%)	19 (34.5%)
	Third	79 (19.8%)	4 (7.3%)
	Fourth	5 (1.3%)	4 (7.3%)
	Unclassified	16 (4.0%)	8 (14.5%)

a Determined according to the corresponding institution.

b All others *n* < 6 including: Australia, Belgium, Egypt, France, Greece, Hong Kong, Hungary, India, Mexico, Nepal, the Netherlands, New Zealand, Pakistan, Spain, Sweden, Switzerland, Taiwan, Thailand, and Vietnam.

c All miscellaneous studies were surveys.

d According to Thomas Reuters Journal Citation Reports.

e All others *n* < 8 including: Anesthiology; Biochemical Research Methods; Biochemistry & Molecular Biology; Biology; Cell Biology; Chemistry, Medicinal; Chemistry, Analytical; Critical Care Medicine; Dentistry, Oral Surgery & Medicine; Dermatology; Electrochemistry; Environmental Sciences; Gastroenterology & Hepatology; Genetics & Heredity; Hematology; Immunology; Medical Laboratory Technology; Medicine, Research & Experimental; Neurosciences; Oncology; Pediatrics; Pharmacology & Pharmacy; Psychiatry; Public, Environmental & Occupational Health; Respiratory System; Surgery; unclassified.

f Ranking according to category‐specific Impact Factor.

### Investigator responsiveness

**Figure 3:**
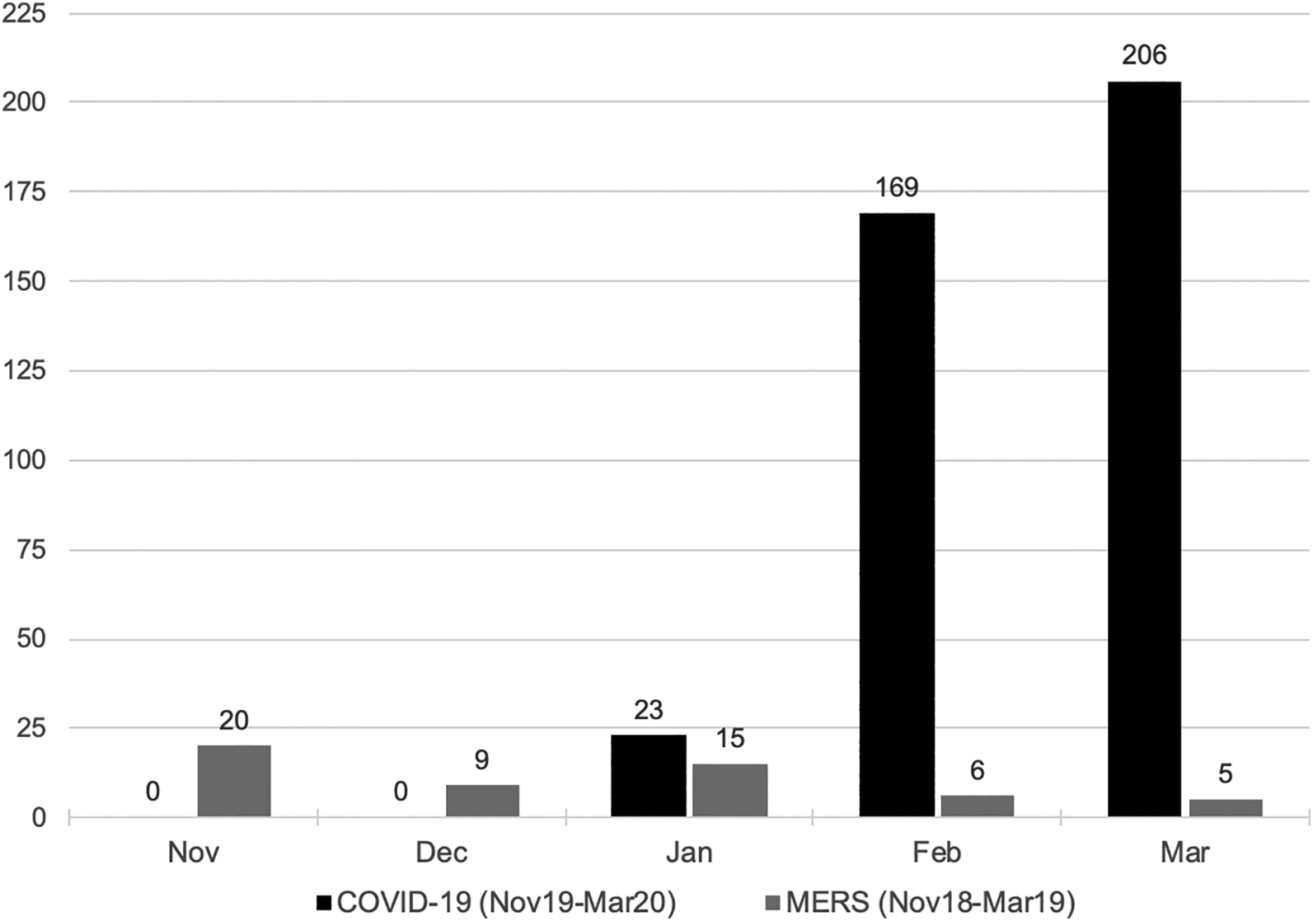
Volume of COVID‐19 and MERS studies by month of publication.

The first eligible COVID‐19 study was reported on 21 January 2020. The volume of reports increased rapidly during the early stages of the disease, with 23 published in January 2020 (5.8%), 169 in February 2020 (*n* = 42.5%), and 206 in March 2020 (51.8%). This compared with a median of nine MERS studies per month (range: 5–20) published during the control period (Fig. 3). The majority of COVID‐19 studies were clinical (*n* = 242; 60.8%), followed by pre‐clinical (*n* = 90; 22.6%), and modelling studies (*n* = 62; 15.6%). Case reports (*n* = 65; 26.9%) and case series (*n* = 105; 43.4%) were the predominant designs for clinical studies and most of these set out to define the disease (*n* = 126; 52.1%). Almost all clinical studies explored general adult populations (*n* = 209; 86.4%), with a handful exploring other groups, including children (*n* = 16; 6.6%), pregnant adults (*n* = 7; 2.9%), and healthcare workers (*n* = 10; 4.1%) (Table 3). In contrast, MERS studies were mostly pre‐clinical (*n* = 37; 67.3%), followed by clinical (*n* = 13; 23.6%), modelling (*n* = 1; 1.8%), and other miscellaneous (*n* = 3; 5.5%). MERS clinical studies mainly comprised of case series and observational designs (both *n* = 5; 38.5%) and most set out to define the disease or explore issues of diagnosis/screening (both *n* = 5; 38.5%). The majority explored general adult populations (*n* = 11, 84.6%).

**Table 3:** Characteristics of clinical COVID‐19 and MERS studies

		COVID‐19 published (*n* = 242)	MERS published (*n* = 13)
Study population	Adults	209 (86.4%)	11 (84.6%)
	Children	16 (6.6%)	0 (0.0%)
	Pregnant adults	7 (2.9%)	0 (0.0%)
	Healthcare workers	10 (4.1%)	2 (15.4%)
Study design	Case report	65 (26.9%)	1 (7.7%)
	Case series	105 (43.4%)	5 (38.5%)
	Observational	64 (26.4%)	5 (38.5%)
	Interventional	5 (2.1%)	2 (15.4%)
		Novel conjunctival secretion RT‐PCR test	–
		Novel rapid IgM–IgG antibody test	–
		Ribavirin, interferon‐alpha	–
		lopinavir/ritonavir	–
		Oxygenation‐assisted tracheal intubation	–
		Lopinavir/ritonavir	–
	Other	3 (1.2%)	0 (0.0%)
Study focus	Definition of disease	126 (52.1%)	5 (38.5%)
	Diagnosis/screening	81 (33.5%)	5 (38.5%)
	Prevention	15 (6.2%)	0 (0.0%)
	Treatment	13 (5.4%)	3 (23.1%)
	Resource use	1 (0.4%)	0 (0.0%)
	Other miscellaneous	6 (2.5%)	0 (0.0%)

RT‐PCR, real‐time reverse transcription polymerase chain reaction.

### Editorial responsiveness

The time taken for editorial and peer review was available for 257 (64.6%) COVID‐19 studies and 52 (94.5%) MERS studies. The median time from submission to acceptance was much shorter for COVID‐19 studies [median: 5 days; interquartile range (IQR): 3–11] compared with MERS (median: 71.5 days; IQR: 38–106) (*P* < .001) (Table 4). The median time for COVID‐19 short communications was shorter than for full texts (4 days, IQR: 2–7.5 vs. 6 days, IQR 3–13; *P* < .001), whereas no significant difference in time was found between study designs (case report: 4.5, IQR 2–10 vs. case series: 5, IQR 2·25–10 vs. observational: 6, IQR 3–13; *P* = .645). Original data were available via a supplement, data repository, or through direct request from the study authors for 104 (26.1%) COVID‐19 studies compared with 10 (18.2%) MERS (*P* = .203). When case reports were excluded, data were available for 95 of 333 (28.5%) COVID‐19 studies and 10 out of 54 (18.5%) MERS studies.

**Table 4:** Editorial and publisher responsiveness

		COVID‐19	MERS
Time to acceptance^a^	Median	5 days	71.5 days
	Interquartile range	3–11	38–106
Time to publication^b^	Median	5 days	22.5 days
	Interquartile range	2–8	4–48.5

a Time from submission to acceptance.

b Time from acceptance to publication.

### Publisher responsiveness

The time taken for production processes was available for 280 (70.4%) COVID‐19 and 52 (94.5%) MERS studies. The median time from acceptance to first publication was significantly shorter for COVID‐19 (5 days, IQR: 2–8) compared with MERS (22.5 days, IQR: 4–48·5; *P* = .001). There was no significant difference in the time for production between letters/short communications (median: 5, IQR: 1.5–8) and full texts (median: 5, IQR: 2–8) (*P* = .617). Neither was there a significant difference in production time between study designs (case report: 5, IQR: 3–8 vs. case series: 5, IQR 2–8 vs. observational: 5, IQR: 3–7·75) (*P* = 0.954). A total of 396 (99.5%) COVID‐19 manuscripts were available with open‐access, including 275 (69.4%) manuscripts published in hybrid journals (open‐access not mandated). Unexpectedly, all 55 (100%) MERS manuscripts were also available with open‐access, including 26 (47.3%) manuscripts published in hybrid journals. The difference in open‐access between the two diseases was not significant (*P* = 1.000).

#### Assessment of quality

An assessment of self‐declared compliance to reporting guidelines was performed for 48 full‐text COVID‐19 studies (*n* = 45 observational; *n* = 3 interventional) and 6 full‐text MERS studies (*n* = 4 observational; *n* = 2 interventional). None of these studies declared compliance to relevant reporting checklists, as recommended by the Equator Network.

## DISCUSSION

The results demonstrate a rapid academic response to COVID‐19 during its early stages. Investigators responded with reports of cases and case series, many of which were published in major general medical journals. The volume of interventional studies exploring treatments was low, but this is expected to change as on‐going trials reach completion. The editorial and production times for COVID‐19 studies were strikingly shorter than MERS controls and almost all manuscripts were openly accessible at the point of publication. In contrast, only one‐in‐four COVID‐19 manuscripts were published with original data available to support the results.

This study raises important considerations for the dissemination of data during the COVID‐19 pandemic. First, the urgency to produce evidence and the need for rigorous peer review must be balanced to ensure quality of published outputs. In this study, an absence of self‐declared compliance to reporting checklists (such as STROBE and CONSORT) may indicate a relaxation of reporting standards. The average time to manuscript acceptance of 5 days may also suggest a pattern of internal editorial review, rather than review by externally invited subject‐matter experts. It is not possible to explore this with certainty from the present data because details of peer review are not usually available. While there is an urgent need to report outputs quickly, other routes for provisional dissemination (such as pre‐prints) may be important to protect the robustness of peer review. Second, open‐access publication is essential so that the global medical community can learn freely from each other. In this study, almost all articles were available with open‐access for both COVID‐19 and MERS research. It is likely that this represents an extended effort by publishers to share knowledge, since only 45% of the scholarly literature in 2015 was published with open‐access (Piwowar *et al*., 2018). Consideration is needed to how this approach can be sustained as the pandemic progresses through its recovery and subsequent outbreak phases. Finally, this study provides an early insight into the challenges of data sharing during the COVID‐19 crisis. As more potentially practice‐changing studies emerge, data sharing will be critical for allowing scrutiny of results, reducing unnecessary duplication, and enabling rigorous pooled analyses. Awareness of secure access data repositories as well as guidelines for data stewardship will be important (Wilkinson *et al*., 2016).

The academic response during times of global disease has been explored previously. In a bibliometric analysis of the 2003 severe acute respiratory syndrome (SARS) crisis, the difference in median submission‐to‐acceptance intervals between SARS and non‐SARS articles was 106.5 days (95% CI 55.0–140.1) and the difference in median acceptance‐to‐publication intervals was 63.5 days (18.0–94.1) (Xing, Hejblum, Leung, & Valleron, 2010). Interestingly, the median time to acceptance (55 days) and publication (77.5 days) of SARS articles was much longer than the corresponding times of COVID‐19 research in the present study. It is possible that this reflects a longer period of study inclusion, spanning both active and non‐active outbreak phases. In another bibliometric review of the West African Ebola Virus Disease (EVD), the academic response was shown to be organized around a small number of individuals with extensive global networks. This demonstrated the role and importance of strategic planning through international cooperation and expertise (Hagel, Weidemann, Gauch, Edwards, & Tinnemann, 2017). More recently, two analyses of COVID‐19 research found that most data were generated from China and called for increased academic output, particularly in the form of interventional trials of new treatments (Chahrour *et al*., 2020; Lou *et al*., 2020). As shown by the previous response to EVD and the current data, it is clear this must be done with global coordination and with a common research agenda (COVID‐19 Clinical Research Coalition, 2020).

Strengths of this study are recognized. This review provides timely and critical information about how the academic community have responded to this global emergency during its early stages. Specifically, it identifies on‐going challenges that must be considered, such as the balance between rapid and robust review, as well as the availability of original data to support published results. Weaknesses are also recognized. This review provides an incomplete representation of the entire research pathway. While investigators, editors, and publishers are essential for generating and disseminating research, other groups such as funding bodies, research ethics committees, regulatory agencies, and patient/stakeholder groups also play important roles. Measuring the response of these groups is challenging since it relies on data that are not openly available or straight forward to measure. Another weakness is the focus on peer‐reviewed publications. It is likely that a wider body of unpublished data exist following rejection or through investigators making results available on non‐peer‐reviewed platforms such as pre‐print servers. These are important and further work to confirm their rigour and stability (i.e. changes made during parallel submission and peer review) is needed.

There is no question that the academic response to COVID‐19 from investigators, editors, and publishers has been prompt. Data sharing is an early challenge and a commitment to this from all members of the academic community is required. Consideration to how high‐quality peer review can be upheld in the face of great pressure to disseminate data must also be considered. As the pandemic develops, it is likely that the anatomy of COVID‐19 research will shift from efforts to describe the disease towards efforts to treat and prevent it. Further auditing of the academic response will be important so that the academic community can identify and address new challenges as they emerge.

## AUTHOR CONTRIBUTIONS

SJC and DGJ conceptualized the study. SJC, JAH and WSB performed searches, study assessments, and data extraction. SJC, JAH, JRB, and JPT prepared the manuscript, which was subsequently edited by all authors. SJC is the study guarantor.

## CONFLICT OF INTEREST

JB holds an RCS England clinical research fellowship supported by Bowel Cancer UK. DGJ is funded by Bowel Cancer UK and RCS England. SJC holds a Doctoral Research Fellowship (DRF‐2018‐11‐049) supported by the National Institute of Health Research. The views expressed in this publication are those if the authors and not necessarily those of the NHS, the National Institute for Health Research, Health Education England, or the Department of Health. This research did not receive any specific grant from funding agencies in the public, commercial, or not‐for‐profit sectors. All coded data will be available at the point of publication. The data and data dictionary will be hosted via Figshare: https://doi.org/10.6084/m9.figshare.12442379. The corresponding author (SJC) attests that all authors had access to study data, takes responsibility for the accuracy of the analysis, and had authority over manuscript preparation and the decision to submit the manuscript for publication.

## Supporting information


**Appendix S1** COVID‐19 study title.

**Appendix S2** MERS study title.
