# Supplementary material for: Global academic response to COVID‐19: Cross‐sectional study
Source: Learn Publ. 2020 Jul 1;33(4):385–93. doi: 10.1002/leap.1317 (PMC7362145; doi:10.1002/leap.1317)
Supplement: Supplementary file 1 — Appendix S1 COVID‐19 study title. [file LEAP-33-385-s001.docx]

| **ID** | **COVID-19 Study Title** |
| --- | --- |
| 1 | Estimated effectiveness of symptom and risk screening to prevent the spread of COVID-19. |
| 2 | Novel coronavirus disease (Covid-19): the first two patients in the UK with person to person transmission. |
| 3 | A family cluster of SARS-CoV-2 infection involving 11 patients in Nanjing, China. |
| 4 | Optimization method for forecasting confirmed cases of COVID-19 in China. |
| 5 | Secondary attack rate and superspreading events for SARS-CoV-2. |
| 6 | Positive rate of RT-PCR detection of SARS-CoV-2 infection in 4880 cases from one hospital in Wuhan, China, from Jan to Feb 2020. |
| 7 | Nonstructural proteins NS7b and NS8 are likely to be phylogenetically associated with evolution of 2019-nCoV. |
| 8 | Prediction of epidemic spread of the 2019 novel coronavirus driven by spring festival transportation in China: A population-based study. |
| 9 | Public's early response to the novel coronavirus-infected pneumonia. |
| 10 | Epidemiologic Features and Clinical Course of Patients Infected with SARS-CoV-2 in Singapore. |
| 11 | Clinical and computed tomographic imaging features of novel coronavirus pneumonia caused by SARS-CoV-2. |
| 12 | Asymptomatic novel coronavirus pneumonia patient outside Wuhan: The value of CT images in the course of the disease. |
| 13 | A mathematical model for simulating the phase-based transmissibility of a novel coronavirus. |
| 14 | Detectable 2019-nCoV viral RNA in blood is a strong indicator for the further clinical severity. |
| 15 | Anti-HCV, nucleotide inhibitors, repurposing against COVID-19. |
| 16 | First case of Coronavirus Disease 2019 (COVID-19) pneumonia in Taiwan. |
| 17 | Clinical and biochemical indexes from 2019-nCoV infected patients linked to viral loads and lung injury. |
| 18 | Evolution of the novel coronavirus from the ongoing Wuhan outbreak and modeling of its spike protein for risk of human transmission. |
| 19 | Feasibility of controlling COVID-19 outbreaks by isolation of cases and contacts. |
| 20 | Backcalculating the incidence of infection with covid-19 on the diamond princess. |
| 21 | A conceptual model for the coronavirus disease 2019 (COVID-19) outbreak in Wuhan, China with individual reaction and governmental action. |
| 22 | Transmission potential of the novel coronavirus (COVID-19) onboard the diamond Princess Cruises Ship, 2020. |
| 23 | Imaging and clinical features of patients with 2019 novel coronavirus SARS-CoV-2. |
| 24 | Development of epitope-based peptide vaccine against novel coronavirus 2019 (SARS-COV-2): Immunoinformatics approach. |
| 25 | Nowcasting and forecasting the potential domestic and international spread of the 2019-nCoV outbreak originating in Wuhan, China: a modelling study. |
| 26 | Estimation of the reproductive number of novel coronavirus (COVID-19) and the probable outbreak size on the Diamond Princess cruise ship: A data-driven analysis. |
| 27 | Distribution of the COVID-19 epidemic and correlation with population emigration from wuhan, China. |
| 28 | The Clinical and Chest CT Features Associated with Severe and Critical COVID-19 Pneumonia. |
| 29 | Persistence and clearance of viral RNA in 2019 novel coronavirus disease rehabilitation patients. |
| 30 | COVID-19 in 2 Persons with Mild Upper Respiratory Symptoms on a Cruise Ship, Japan. |
| 31 | Analysis of factors associated with disease outcomes in hospitalized patients with 2019 novel coronavirus disease. |
| 32 | Trend and forecasting of the COVID-19 outbreak in China. |
| 33 | Comparison of different samples for 2019 novel coronavirus detection by nucleic acid amplification tests. |
| 34 | Single-cell RNA sequencing data suggest a role for angiotensin-converting enzyme 2 in kidney impairment in patients infected with 2019-nCoV. |
| 35 | A case of 2019 Novel Coronavirus in a pregnant woman with preterm delivery. |
| 36 | A Chinese Case of COVID-19 Did Not Show Infectivity During the Incubation Period: Based on an Epidemiological Survey. |
| 37 | COVID-19: combining antiviral and anti-inflammatory treatments. |
| 38 | Pulmonary pathology of early phase 2019 novel coronavirus (COVID-19) pneumonia in two patients with lung cancer. |
| 39 | Clinical Characteristics of Imported Cases of COVID-19 in Jiangsu Province: A Multicenter Descriptive Study. |
| 40 | COVID-19 outbreak on the Diamond Princess cruise ship: estimating the epidemic potential and effectiveness of public health countermeasures. |
| 41 | Characteristics of COVID-19 infection in Beijing. |
| 42 | Early phylogenetic estimate of the effective reproduction number of SARS-CoV-2. |
| 43 | Immunoinformatics-aided identification of T cell and B cell epitopes in the surface glycoprotein of 2019-nCoV. |
| 44 | First imported case of 2019 novel coronavirus in Canada, presenting as mild pneumonia. |
| 45 | Comparative genetic analysis of the novel coronavirus (2019-nCoV/SARS-CoV-2) receptor ACE2 in different populations. |
| 46 | Clinical course and outcomes of critically ill patients with SARS-CoV-2 pneumonia in Wuhan, China: a single-centered, retrospective, observational study. |
| 47 | Negative Nasopharyngeal and Oropharyngeal Swab Does Not Rule Out COVID-19. |
| 48 | Clinical characteristics of 140 patients infected with SARS-CoV-2 in Wuhan, China. |
| 49 | The first 2019 novel coronavirus case in Nepal. |
| 50 | Development and Clinical Application of A Rapid IgM-IgG Combined Antibody Test for SARS-CoV-2 Infection Diagnosis. |
| 51 | Nepal's First Case of COVID-19 and public health response. |
| 52 | Systematic Comparison of Two Animal-to-Human Transmitted Human Coronaviruses: SARS-CoV-2 and SARS-CoV. |
| 53 | 2019 Novel Coronavirus (COVID-19) Pneumonia: Serial Computer Tomography Findings. |
| 54 | Evaluation of coronavirus in tears and conjunctival secretions of patients with SARS-CoV-2 infection. |
| 55 | Evolutionary history, potential intermediate animal host, and cross-species analyses of SARS-CoV-2. |
| 56 | Positive RT-PCR Test Results in Patients Recovered From COVID-19. |
| 57 | Correlation of Chest CT and RT-PCR Testing in Coronavirus Disease 2019 (COVID-19) in China: A Report of 1014 Cases. |
| 58 | A novel coronavirus from patients with pneumonia in China, 2019. |
| 59 | Cancer patients in SARS-CoV-2 infection: a nationwide analysis in China. |
| 60 | Epidemiological identification of a novel pathogen in real time: Analysis of the atypical pneumonia outbreak in Wuhan, China, 2019-2020. |
| 61 | Clinical characteristics of laboratory confirmed positive cases of SARS-CoV-2 infection in Wuhan, China: A retrospective single center analysis. |
| 62 | Assessing the impact of reduced travel on exportation dynamics of novel coronavirus infection (Covid-19). |
| 63 | Cross-species transmission of the newly identified coronavirus 2019-nCoV. |
| 64 | Genetic diversity and evolution of SARS-CoV-2. |
| 65 | Composition and divergence of coronavirus spike proteins and host ACE2 receptors predict potential intermediate hosts of SARS-CoV-2. |
| 66 | Passengers' destinations from China: low risk of Novel Coronavirus (2019-nCoV) transmission into Africa and South America. |
| 67 | Analysis of angiotensin-converting enzyme 2 (ACE2) from different species sheds some light on cross-species receptor usage of a novel coronavirus 2019-nCoV. |
| 68 | Chest Radiographic and CT Findings of the 2019 Novel Coronavirus Disease (COVID-19): Analysis of Nine Patients Treated in Korea. |
| 69 | Imported cases of 2019-novel coronavirus (2019-nCoV) infections in Thailand: Mathematical modelling of the outbreak. |
| 70 | Dose prediction of lopinavir/ritonavir for 2019-novel coronavirus (2019-nCoV) infection based on mathematic modeling. |
| 71 | Does SARS-CoV-2 has a longer incubation period than SARS and MERS?. |
| 72 | Novel coronavirus pneumonia outbreak in 2019: Computed tomographic findings in two cases. |
| 73 | Short-term forecasts of the COVID-19 epidemic in Guangdong and Zhejiang, China: February 13-23, 2020. |
| 74 | Genomic characterisation and epidemiology of 2019 novel coronavirus: implications for virus origins and receptor binding. |
| 75 | Functional assessment of cell entry and receptor usage for SARS-CoV-2 and other lineage B betacoronaviruses. |
| 76 | Epitopes for a 2019-nCoV vaccine. |
| 77 | High expression of ACE2 receptor of 2019-nCoV on the epithelial cells of oral mucosa. |
| 78 | Novel coronavirus (2019-nCoV) cases in Hong Kong and implications for further spread. |
| 79 | Clinical Features of Atypical 2019 Novel Coronavirus Pneumonia with an initially Negative RT-PCR Assay. |
| 80 | Potential Presymptomatic Transmission of SARS-CoV-2, Zhejiang Province, China, 2020. |
| 81 | Chest CT Findings in Patients with Corona Virus Disease 2019 and its Relationship with Clinical Features. |
| 82 | 2019 novel coronavirus disease (COVID-19) in Taiwan: Reports of two cases from Wuhan, China. |
| 83 | 2019-nCoV (Wuhan virus), a novel Coronavirus: Human-to-human transmission, travel-related cases, and vaccine readiness. |
| 84 | The 2019-new coronavirus epidemic: Evidence for virus evolution. |
| 85 | Preparedness and vulnerability of African countries against importations of COVID-19: a modelling study. |
| 86 | Pathological findings of COVID-19 associated with acute respiratory distress syndrome. |
| 87 | 18F-FDG PET/CT findings of COVID-19: a series of four highly suspected cases. |
| 88 | First Atypical case of 2019 novel coronavirus in Yan'an, China. |
| 89 | Asymptomatic cases in a family cluster with SARS-CoV-2 infection. |
| 90 | The first Vietnamese case of COVID-19 acquired from China. |
| 91 | Phase-adjusted estimation of the number of Coronavirus Disease 2019 cases in Wuhan, China. |
| 92 | Early epidemiological analysis of the coronavirus disease 2019 outbreak based on crowdsourced data: a population-level observational study. |
| 93 | Structure analysis of the receptor binding of 2019-nCoV. |
| 94 | Quantifying the association between domestic travel and the exportation of novel coronavirus (2019-nCoV) cases from Wuhan, China in 2020: A correlational analysis. |
| 95 | Presumed Asymptomatic Carrier Transmission of COVID-19. |
| 96 | Viral Load Kinetics of SARS-CoV-2 Infection in First Two Patients in Korea. |
| 97 | Virus Isolation from the First Patient with SARS-CoV-2 in Korea. |
| 98 | Cryo-EM structure of the 2019-nCoV spike in the prefusion conformation. |
| 99 | Detection of 2019 novel coronavirus (2019-nCoV) by real-time RT-PCR. |
| 100 | Potential of large "first generation" human-to-human transmission of 2019-nCoV. |
| 101 | SARS-CoV-2 Viral Load in Upper Respiratory Specimens of Infected Patients. |
| 102 | Development of Genetic Diagnostic Methods for Novel Coronavirus 2019 (nCoV-2019) in Japan. |
| 103 | Chest CT Findings in Coronavirus Disease-19 (COVID-19): Relationship to Duration of Infection. |
| 104 | Genomic variance of the 2019-nCoV coronavirus. |
| 105 | Imaging features of 2019 novel coronavirus pneumonia. |
| 106 | Novel Coronavirus Infection in Hospitalized Infants under 1 Year of Age in China. |
| 107 | RNA based mNGS approach identifies a novel human coronavirus from two individual pneumonia cases in 2019 Wuhan outbreak. |
| 108 | A familial cluster of pneumonia associated with the 2019 novel coronavirus indicating person-to-person transmission: a study of a family cluster. |
| 109 | Laboratory readiness and response for novel coronavirus (2019-nCoV) in expert laboratories in 30 EU/EEA countries, January 2020. |
| 110 | Risk assessment of novel coronavirus COVID-19 outbreaks outside China. |
| 111 | Sensitivity of Chest CT for COVID-19: Comparison to RT-PCR. |
| 112 | Abnormal Coagulation parameters are associated with poor prognosis in patients with novel coronavirus pneumonia. |
| 113 | Changes of CT Findings in a 2019 Novel Coronavirus (2019-nCoV) pneumonia patient. |
| 114 | First cases of coronavirus disease 2019 (COVID-19) in France: surveillance, investigations and control measures, January 2020. |
| 115 | A familial cluster of infection associated with the 2019 novel coronavirus indicating potential person-to-person transmission during the incubation period. |
| 116 | Novel coronavirus outbreak in Wuhan, China, 2020: Intense surveillance is vital for preventing sustained transmission in new locations. |
| 117 | Epidemiological and clinical characteristics of 99 cases of 2019 novel coronavirus pneumonia in Wuhan, China: a descriptive study. |
| 118 | Real-time estimation of the risk of death from novel coronavirus (COVID-19) infection: Inference using exported cases. |
| 119 | Incubation period and other epidemiological characteristics of 2019 novel coronavirus infections with right truncation: A statistical analysis of publicly available case data. |
| 120 | Molecular and serological investigation of 2019-nCoV infected patients: implication of multiple shedding routes. |
| 121 | Potent binding of 2019 novel coronavirus spike protein by a SARS coronavirus-specific human monoclonal antibody. |
| 122 | HIV-1 did not contribute to the 2019-nCoV genome. |
| 123 | Initial CT findings and temporal changes in patients with the novel coronavirus pneumonia (2019-nCoV): a study of 63 patients in Wuhan, China. |
| 124 | Preliminary estimation of the basic reproduction number of novel coronavirus (2019-nCoV) in China, from 2019 to 2020: A data-driven analysis in the early phase of the outbreak. |
| 125 | An updated estimation of the risk of transmission of the novel coronavirus (2019-nCov). |
| 126 | The spike glycoprotein of the new coronavirus 2019-nCoV contains a furin-like cleavage site absent in CoV of the same clade. |
| 127 | Clinical features of patients infected with 2019 novel coronavirus in Wuhan, China. |
| 128 | Real-time forecasts of the COVID-19 epidemic in China from February 5th to February 24th, 2020. |
| 129 | The first two cases of 2019-nCoV in Italy: Where they come from?. |
| 130 | Estimation of the transmission risk of the 2019-nCoV and its implication for public health interventions. |
| 131 | Case of the Index Patient Who Caused Tertiary Transmission of COVID-19 Infection in Korea: the Application of Lopinavir/Ritonavir for the Treatment of COVID-19 Infected Pneumonia |
| 132 | The global spread of 2019-nCoV: a molecular evolutionary analysis. |
| 133 | The rate of underascertainment of novel coronavirus (2019-ncov) infection: Estimation using japanese passengers data on evacuation flights. |
| 134 | A Locally Transmitted Case of SARS-CoV-2 Infection in Taiwan. |
| 135 | Journey of a Thai Taxi Driver and Novel Coronavirus. |
| 136 | Pre- and Posttreatment Chest CT Findings: 2019 Novel Coronavirus (2019-nCoV) Pneumonia. |
| 137 | Chest CT for Typical 2019-nCoV Pneumonia: Relationship to Negative RT-PCR Testing. |
| 138 | Use of Chest CT in Combination with Negative RT-PCR Assay for the 2019 Novel Coronavirus but High Clinical Suspicion. |
| 139 | Fusion mechanism of 2019-nCoV and fusion inhibitors targeting HR1 domain in spike protein. |
| 140 | Effectiveness of airport screening at detecting travellers infected with novel coronavirus (2019-nCoV). |
| 141 | Risk for Transportation of 2019 Novel Coronavirus Disease from Wuhan to Other Cities in China. |
| 142 | Time Course of Lung Changes On Chest CT During Recovery From 2019 Novel Coronavirus (COVID-19) Pneumonia. |
| 143 | Consistent detection of 2019 novel coronavirus in saliva. |
| 144 | Incubation period of 2019 novel coronavirus (2019-nCoV) infections among travellers from Wuhan, China, 20-28 January 2020. |
| 145 | Transmission dynamics and evolutionary history of 2019-nCoV. |
| 146 | Preliminary prediction of the basic reproduction number of the Wuhan novel coronavirus 2019-nCoV. |
| 147 | Clinical Features and Treatment of 2019-nCov Pneumonia Patients in Wuhan: Report of A Couple Cases. |
| 148 | Clinical characteristics and intrauterine vertical transmission potential of COVID-19 infection in nine pregnant women: a retrospective review of medical records. |
| 149 | Clinical characteristics of novel coronavirus cases in tertiary hospitals in Hubei Province. |
| 150 | Epidemiologic and Clinical Characteristics of Novel Coronavirus Infections Involving 13 Patients Outside Wuhan, China. |
| 151 | Clinical Characteristics of 138 Hospitalized Patients with 2019 Novel Coronavirus-Infected Pneumonia in Wuhan, China. |
| 152 | Genome Composition and Divergence of the Novel Coronavirus (2019-nCoV) Originating in China. |
| 153 | Evolution of CT Manifestations in a Patient Recovered from 2019 Novel Coronavirus (2019-nCoV) Pneumonia in Wuhan, China. |
| 154 | Clinical characteristics and therapeutic procedure for four cases with 2019 novel coronavirus pneumonia receiving combined Chinese and Western medicine treatment. |
| 155 | Epidemiologic characteristics of early cases with 2019 novel coronavirus (2019-nCoV) disease in Republic of Korea. |
| 156 | Emerging Coronavirus 2019-nCoV Pneumonia. |
| 157 | The Outbreak Cases with the Novel Coronavirus Suggest Upgraded Quarantine and Isolation in Korea. |
| 158 | The First Case of 2019 Novel Coronavirus Pneumonia Imported into Korea from Wuhan, China: Implication for Infection Prevention and Control Measures. |
| 159 | CT Manifestations of Two Cases of 2019 Novel Coronavirus (2019-nCoV) Pneumonia. |
| 160 | Molecular Diagnosis of a Novel Coronavirus (2019-nCoV) Causing an Outbreak of Pneumonia. |
| 161 | Reporting, Epidemic Growth, and Reproduction Numbers for the 2019 Novel Coronavirus (2019-nCoV) Epidemic. |
| 162 | Estimating the unreported number of novel coronavirus (2019-ncov) cases in China in the first half of january 2020: A data-driven modelling analysis of the early outbreak. |
| 163 | Novel coronavirus (2019-nCoV) early-stage importation risk to Europe, January 2020. |
| 164 | CT Imaging Features of 2019 Novel Coronavirus (2019-nCoV). |
| 165 | Pattern of early human-to-human transmission of Wuhan 2019 novel coronavirus (2019-nCoV), December 2019 to January 2020. |
| 166 | Remdesivir and chloroquine effectively inhibit the recently emerged novel coronavirus (2019-nCoV) in vitro. |
| 167 | Full-genome evolutionary analysis of the novel corona virus (2019-nCoV) rejects the hypothesis of emergence as a result of a recent recombination event. |
| 168 | A pneumonia outbreak associated with a new coronavirus of probable bat origin. |
| 169 | 2019 Novel Coronavirus (2019-nCoV) Pneumonia. |
| 170 | First Case of 2019 Novel Coronavirus in the United States. |
| 171 | Identification of a novel coronavirus causing severe pneumonia in human: a descriptive study. |
| 172 | CT Imaging of the 2019 Novel Coronavirus (2019-nCoV) Pneumonia. |
| 173 | Transmission of 2019-nCoV Infection from an Asymptomatic Contact in Germany. |
| 174 | Early Transmission Dynamics in Wuhan, China, of Novel Coronavirus-Infected Pneumonia. |
| 175 | Receptor recognition by novel coronavirus from Wuhan: An analysis based on decade-long structural studies of SARS. |
| 176 | Genomic characterization of the 2019 novel human-pathogenic coronavirus isolated from a patient with atypical pneumonia after visiting Wuhan. |
| 177 | Successful recovery of COVID-19 pneumonia in a renal transplant recipient with long-term immunosuppression. |
| 178 | Diagnostic Utility of Clinical Laboratory Data Determinations for Patients with the Severe COVID-19. |
| 179 | Platelet-to-lymphocyte ratio is associated with prognosis in patients with Corona Virus Disease-19. |
| 180 | Epidemiologic and Clinical Characteristics of 91 Hospitalized Patients with COVID-19 in Zhejiang, China: A retrospective, multi-centre case series. |
| 181 | Early Clinical and CT Manifestations of Coronavirus Disease 2019 (COVID-19) Pneumonia. |
| 182 | The positive impact of lockdown in Wuhan on containing the COVID-19 outbreak in China. |
| 183 | Correlation between travellers departing from Wuhan before the Spring Festival and subsequent spread of COVID-19 to all provinces in China. |
| 184 | Safety and efficacy of different anesthetic regimens for parturients with COVID-19 undergoing Cesarean delivery: a case series of 17 patients. |
| 185 | Clinical features of pediatric patients with COVID-19: a report of two family cluster cases. |
| 186 | Clinical outcome of 55 asymptomatic cases at the time of hospital admission infected with SARS-Coronavirus-2 in Shenzhen, China. |
| 187 | A 55-Day-Old Female Infant infected with COVID 19: presenting with pneumonia, liver injury, and heart damage. |
| 188 | Risk Factors of Healthcare Workers with Corona Virus Disease 2019: A Retrospective Cohort Study in a Designated Hospital of Wuhan in China. |
| 189 | Substantial undocumented infection facilitates the rapid dissemination of novel coronavirus (SARS-CoV2). |
| 190 | Clinical features and dynamics of viral load in imported and non-imported patients with COVID-19. |
| 191 | Testing the repatriated for SARS-Cov2: Should laboratory-based quarantine replace traditional quarantine?. |
| 192 | Clinical diagnostic value of CT imaging in COVID-19 with multiple negative RT-PCR testing. |
| 193 | Composition of human-specific slow codons and slow di-codons in SARS-CoV and 2019-nCoV are lower than other coronaviruses suggesting a faster protein synthesis rate of SARS-CoV and 2019 nCoV |
| 194 | Anesthetic Management of Patients With Suspected or Confirmed 2019 Novel Coronavirus Infection During Emergency Procedures. |
| 195 | First known person-to-person transmission of severe acute respiratory syndrome coronavirus 2 (SARS-CoV-2) in the USA. |
| 196 | Clinical Features of 69 Cases with Coronavirus Disease 2019 in Wuhan, China. |
| 197 | Prominent changes in blood coagulation of patients with SARS-CoV-2 infection. |
| 198 | Genetic evolution analysis of 2019 novel coronavirus and coronavirus from other species. |
| 199 | Evidence of the COVID-19 Virus Targeting the CNS: Tissue Distribution, Host-Virus Interaction, and Proposed Neurotropic Mechanisms. |
| 200 | Risk Factors Associated With Acute Respiratory Distress Syndrome and Death in Patients With Coronavirus Disease 2019 Pneumonia in Wuhan, China. |
| 201 | Detection of SARS-CoV-2 in Different Types of Clinical Specimens. |
| 202 | Traditional Chinese medicine for COVID-19 treatment. |
| 203 | Air, Surface Environmental, and Personal Protective Equipment Contamination by Severe Acute Respiratory Syndrome Coronavirus 2 (SARS-CoV-2) From a Symptomatic Patient. |
| 204 | 2019-novel Coronavirus severe adult respiratory distress syndrome in two cases in Italy: An uncommon radiological presentation. |
| 205 | Estimation of Coronavirus Disease 2019 (COVID-19) Burden and Potential for International Dissemination of Infection From Iran. |
| 206 | COVID-19 and the Risk to Health Care Workers: A Case Report. |
| 207 | Clinical Features and Chest CT Manifestations of Coronavirus Disease 2019 (COVID-19) in a Single-Center Study in Shanghai, China. |
| 208 | 2019 Novel Coronavirus (COVID-19) Pneumonia with Hemoptysis as the Initial Symptom: CT and Clinical Features. |
| 209 | Evolution of Computed Tomography Manifestations in Five Patients Who Recovered from Coronavirus Disease 2019 (COVID-19) Pneumonia. |
| 210 | False-Negative Results of Real-Time Reverse-Transcriptase Polymerase Chain Reaction for Severe Acute Respiratory Syndrome Coronavirus 2: Role of Deep-Learning-Based CT: Diagnosis and Insights from Two Cases. |
| 211 | COVID-19 and mycoplasma pneumoniae coinfection. |
| 212 | Clinical characteristics of refractory COVID-19 pneumonia in Wuhan, China. |
| 213 | Patients of COVID-19 may benefit from sustained lopinavir-combined regimen and the increase of eosinophil may predict the outcome of COVID-19 progression. |
| 214 | Experience of different upper respiratory tract sampling strategies for detection of COVID-19. |
| 215 | Potential inhibitors against 2019-nCoV coronavirus M protease from clinically approved medicines. |
| 216 | Preliminary estimation of the novel coronavirus disease (COVID-19) cases in Iran: A modelling analysis based on overseas cases and air travel data. |
| 217 | The effectiveness of quarantine and isolation determine the trend of the COVID-19 epidemics in the final phase of the current outbreak in China. |
| 218 | Arbidol combined with LPV/r versus LPV/r alone against Corona Virus Disease 2019:a retrospective cohort study. [Review] |
| 219 | Clinical progression of patients with COVID-19 in Shanghai, China. |
| 220 | Clinical characteristics of severe acute respiratory syndrome coronavirus 2 reactivation. |
| 221 | Clinical feature of COVID-19 in elderly patients: a comparison with young and middle-aged patients. [Review] |
| 222 | Clinical and CT Imaging Features of the COVID-19 Pneumonia: Focus on Pregnant Women and Children. |
| 223 | Skin damage and the risk of infection among healthcare workers managing coronavirus disease-2019. |
| 224 | Identification of potential cross-protective epitope between a new type of coronavirus (2019-nCoV) and severe acute respiratory syndrome virus. |
| 225 | Real estimates of mortality following COVID-19 infection. |
| 226 | SARS-CoV-2 RNA more readily detected in induced sputum than in throat swabs of convalescent COVID-19 patients. |
| 227 | Clinical course and risk factors for mortality of adult inpatients with COVID-19 in Wuhan, China: a retrospective cohort study. |
| 228 | Vicarious traumatization in the general public, members, and non-members of medical teams aiding in COVID-19 control. |
| 229 | A report of clinical diagnosis and treatment of nine cases of coronavirus disease 2019. |
| 230 | From SARS and MERS CoVs to SARS-CoV-2: Moving toward more biased codon usage in viral structural and nonstructural genes. |
| 231 | Identification of coronavirus sequences in carp cDNA from Wuhan, China. |
| 232 | Transmission dynamics of the COVID-19 outbreak and effectiveness of government interventions: A data-driven analysis. |
| 233 | Simulating and forecasting the cumulative confirmed cases of SARS-CoV-2 in china by Boltzmann function-based regression analyses. |
| 234 | Early dynamics of transmission and control of COVID-19: a mathematical modelling study. |
| 235 | Co-infections of SARS-CoV-2 with multiple common respiratory pathogens in infected patients. |
| 236 | Single-cell RNA-seq data analysis on the receptor ACE2 expression reveals the potential risk of different human organs vulnerable to 2019-nCoV infection. |
| 237 | Combination of western medicine and Chinese traditional patent medicine in treating a family case of COVID-19 in Wuhan. |
| 238 | Impact of international travel and border control measures on the global spread of the novel 2019 coronavirus outbreak. |
| 239 | COVID-19 Spike-host cell receptor GRP78 binding site prediction. |
| 240 | Epidemiology of 2019 Novel Coronavirus Disease-19 in Gansu Province, China, 2020. |
| 241 | Estimating Risk for Death from 2019 Novel Coronavirus Disease, China, January-February 2020. |
| 242 | Case-Fatality Risk Estimates for COVID-19 Calculated by Using a Lag Time for Fatality. |
| 243 | A Diabetic Patient With 2019-nCoV Infection Who Recovered and Was Discharged From Hospital. |
| 244 | Initial clinical features of suspected Coronavirus Disease 2019 in two emergency departments outside of Hubei, China. |
| 245 | The establishment of reference sequence for SARS-CoV-2 and variation analysis. |
| 246 | The potential chemical structure of anti-SARS-CoV-2 RNA-dependent RNA polymerase. |
| 247 | An exclusive 42 amino acid signature in pp1ab protein provides insights into the evolutive history of the 2019 novel human-pathogenic coronavirus (SARS-CoV2). |
| 248 | A Novel Approach for a Novel Pathogen: using a home assessment team to evaluate patients for 2019 novel coronavirus (SARS-CoV-2). |
| 249 | Enhanced isolation of SARS-CoV-2 by TMPRSS2-expressing cells. |
| 250 | Atypical lung feature on chest CT in a lung adenocarcinoma cancer patient infected with COVID-19. |
| 251 | COVID-19 with spontaneous pneumomediastinum. |
| 252 | Estimating the reproductive number and the outbreak size of Novel Coronavirus disease (COVID-19) using mathematical model in Republic of Korea. |
| 253 | SARS-CoV-2 Infection among Travelers Returning from Wuhan, China. |
| 254 | Detection of Covid-19 in Children in Early January 2020 in Wuhan, China. |
| 255 | Histopathologic Changes and SARS-CoV-2 Immunostaining in the Lung of a Patient With COVID-19. |
| 256 | The transmission and diagnosis of 2019 novel coronavirus infection disease (COVID-19): A Chinese perspective. |
| 257 | Fecal specimen diagnosis 2019 novel coronavirus-infected pneumonia. |
| 258 | Indirect Virus Transmission in Cluster of COVID-19 Cases, Wenzhou, China, 2020. |
| 259 | Rapid Identification of Potential Inhibitors of SARS-CoV-2 Main Protease by Deep Docking of 1.3 Billion Compounds. |
| 260 | Chest computed tomography images of early coronavirus disease (COVID-19). |
| 261 | Chest computed tomography in children with COVID-19 respiratory infection. |
| 262 | A comparative study on the clinical features of COVID-19 pneumonia to other pneumonias. |
| 263 | A case report of neonatal COVID-19 infection in China. |
| 264 | Dysregulation of immune response in patients with COVID-19 in Wuhan, China. |
| 265 | Positive result of Sars-Cov-2 in sputum from a cured patient with COVID-19 |
| 266 | Co-infection of SARS-CoV-2 and HIV in a patient in Wuhan city, China. |
| 267 | Severe Acute Respiratory Syndrome Coronavirus 2 from Patient with 2019 Novel Coronavirus Disease, United States. |
| 268 | Co-infection with SARS-CoV-2 and Influenza A Virus in Patient with Pneumonia, China. |
| 269 | Nepal's First Case of COVID-19 and public health response. |
| 270 | CT Manifestations of Novel Coronavirus Pneumonia: A Case Report. |
| 271 | Performance of radiologists in differentiating COVID-19 from viral pneumonia on chest CT. |
| 272 | In Vitro Antiviral Activity and Projection of Optimized Dosing Design of Hydroxychloroquine for the Treatment of Severe Acute Respiratory Syndrome Coronavirus 2 (SARS-CoV-2). |
| 273 | Detection of Novel Coronavirus by RT-PCR in Stool Specimen from Asymptomatic Child, China. |
| 274 | Clinical characteristics and imaging manifestations of the 2019 novel coronavirus disease (COVID-19):A multi-center study in Wenzhou city, Zhejiang, China. |
| 275 | Comparative effectiveness and safety of ribavirin plus interferon-alpha, lopinavir/ritonavir plus interferon-alpha and ribavirin plus lopinavir/ritonavir plus interferon-alphain in patients with mild to moderate novel coronavirus pneumonia. |
| 276 | High-throughput sequencing for confirmation of suspected 2019-nCoV infection identified by fluorescence quantitative polymerase chain reaction. |
| 277 | Repurposing of clinically approved drugs for treatment of coronavirus disease 2019 in a 2019-novel coronavirus (2019-nCoV) related coronavirus model. |
| 278 | A confirmed asymptomatic carrier of 2019 novel coronavirus (SARS-CoV-2). |
| 279 | Diagnosis and Management of First Case of COVID-19 in Canada: Lessons applied from SARS. |
| 280 | The Treatment and Outcome of a Lung Cancer Patient Infected with SARS-CoV-2. |
| 281 | Recurrence of positive SARS-CoV-2 RNA in COVID-19: A case report. |
| 282 | Clinical characteristics of 24 asymptomatic infections with COVID-19 screened among close contacts in Nanjing, China. |
| 283 | Clinical manifestations and outcome of SARS-CoV-2 infection during pregnancy. |
| 284 | Covert COVID-19 and false-positive dengue serology in Singapore. |
| 285 | Outbreak investigation for COVID-19 in northern Vietnam. |
| 286 | What can early Canadian experience screening for COVID-19 teach us about how to prepare for a pandemic?. |
| 287 | Clinical and CT imaging features of 2019 novel coronavirus disease (COVID-19). |
| 288 | Effective strategies to prevent coronavirus disease-2019 (COVID-19) outbreak in hospital. |
| 289 | Association between 2019-nCoV transmission and N95 respirator use. |
| 290 | Evidence for gastrointestinal infection of SARS-CoV-2. |
| 291 | SARS-CoV-2 Cell Entry Depends on ACE2 and TMPRSS2 and Is Blocked by a Clinically Proven Protease Inhibitor. |
| 292 | FDG PET/CT of COVID-19. |
| 293 | Clinical and High-Resolution CT Features of the COVID-19 Infection: Comparison of the Initial and Follow-up Changes. |
| 294 | The clinical dynamics of 18 cases of COVID-19 outside of Wuhan, China. |
| 295 | Epidemiological characteristics of 2019-ncoV infections in Shaanxi, China by February 8, 2020. |
| 296 | CT Features of Coronavirus Disease 2019 (COVID-19) Pneumonia in 62 Patients in Wuhan, China. |
| 297 | Lack of Vertical Transmission of Severe Acute Respiratory Syndrome Coronavirus 2, China. |
| 298 | Clinical and CT features in pediatric patients with COVID-19 infection: Different points from adults. |
| 299 | Novel antibody epitopes dominate the antigenicity of spike glycoprotein in SARS-CoV-2 compared to SARS-CoV. |
| 300 | Improved molecular diagnosis of COVID-19 by the novel, highly sensitive and specific COVID-19-RdRp/Hel real-time reverse transcription-polymerase chain reaction assay validated in vitro and with clinical specimens. |
| 301 | Structural basis for the recognition of the SARS-CoV-2 by full-length human ACE2. |
| 302 | Escalating infection control response to the rapidly evolving epidemiology of the Coronavirus disease 2019 (COVID-19) due to SARS-CoV-2 in Hong Kong. |
| 303 | 2019 novel coronavirus is undergoing active recombination. |
| 304 | Coronavirus Disease 2019 (COVID-19): Role of Chest CT in Diagnosis and Management. |
| 305 | Relation Between Chest CT Findings and Clinical Conditions of Coronavirus Disease (COVID-19) Pneumonia: A Multicenter Study. |
| 306 | Genomic diversity of SARS-CoV-2 in Coronavirus Disease 2019 patients. |
| 307 | CT Imaging and Differential Diagnosis of COVID-19. |
| 308 | Hematologic parameters in patients with COVID-19 infection. |
| 309 | Imaging changes in severe COVID-19 pneumonia. |
| 310 | Clinical predictors of mortality due to COVID-19 based on an analysis of data of 150 patients from Wuhan, China. |
| 311 | Community Transmission of Severe Acute Respiratory Syndrome Coronavirus 2, Shenzhen, China, 2020. |
| 312 | Imaging changes of severe COVID-19 pneumonia in advanced stage. |
| 313 | A Well Infant with Coronavirus Disease 2019 (COVID-19) with High Viral Load. |
| 314 | A Case Series of children with 2019 novel coronavirus infection: clinical and epidemiological features. |
| 315 | 2019_nCoV/SARS-CoV-2: rapid classification of betacoronaviruses and identification of Traditional Chinese Medicine as potential origin of zoonotic coronaviruses. |
| 316 | Clinical Characteristics of Coronavirus Disease 2019 in China. |
| 317 | Genome Detective Coronavirus Typing Tool for rapid identification and characterization of novel coronavirus genomes. |
| 318 | Viral load of SARS-CoV-2 in clinical samples. |
| 319 | Radiological findings from 81 patients with COVID-19 pneumonia in Wuhan, China: a descriptive study. |
| 320 | Emerging WuHan (COVID-19) coronavirus: glycan shield and structure prediction of spike glycoprotein and its interaction with human CD26. |
| 321 | Potential impact of seasonal forcing on a SARS-CoV-2 pandemic. |
| 322 | Complete Genome Sequence of a 2019 Novel Coronavirus (SARS-CoV-2) Strain Isolated in Nepal. |
| 323 | Early transmission patterns of coronavirus disease 2019 (COVID-19) in travellers from Wuhan to Thailand, January 2020. |
| 324 | Differential diagnosis of illness in patients under investigation for the novel coronavirus (SARS-CoV-2), Italy, February 2020. |
| 325 | The course of clinical diagnosis and treatment of a case infected with coronavirus disease 2019. |
| 326 | Early estimation of the case fatality rate of COVID-19 in mainland China: a data-driven analysis. |
| 327 | Era of molecular diagnosis for pathogen identification of unexplained pneumonia, lessons to be learned. |
| 328 | Rapid establishment of laboratory diagnostics for the novel coronavirus SARS-CoV-2 in Bavaria, Germany, February 2020. |
| 329 | First cases of coronavirus disease 2019 (COVID-19) in the WHO European Region, 24 January to 21 February 2020. |
| 330 | Evaluation of a quantitative RT-PCR assay for the detection of the emerging coronavirus SARS-CoV-2 using a high throughput system. |
| 331 | Immediate Psychological Responses and Associated Factors during the Initial Stage of the 2019 Coronavirus Disease (COVID-19) Epidemic among the General Population in China. |
| 332 | Preliminary Identification of Potential Vaccine Targets for the COVID-19 Coronavirus (SARS-CoV-2) Based on SARS-CoV Immunological Studies. |
| 333 | A new coronavirus associated with human respiratory disease in China. |
| 334 | First two months of the 2019 Coronavirus Disease (COVID-19) epidemic in China: real-time surveillance and evaluation with a second derivative model. |
| 335 | Clinical analysis of 10 neonates born to mothers with 2019-nCoV pneumonia. |
| 336 | Identification of Coronavirus Isolated from a Patient in Korea with COVID-19. |
| 337 | Early Epidemiological and Clinical Characteristics of 28 Cases of Coronavirus Disease in South Korea. |
| 338 | Potential benefits of precise corticosteroids therapy for severe 2019-nCoV pneumonia. |
| 339 | Clinical findings in a group of patients infected with the 2019 novel coronavirus (SARS-Cov-2) outside of Wuhan, China: retrospective case series. |
| 340 | The Effects of Social Support on Sleep Quality of Medical Staff Treating Patients with Coronavirus Disease 2019 (COVID-19) in January and February 2020 in China. |
| 341 | Importation and Human-to-Human Transmission of a Novel Coronavirus in Vietnam. |
| 342 | Clinical findings in a group of patients infected with the 2019 novel coronavirus (SARS-Cov-2) outside of Wuhan, China: retrospective case series. |
| 343 | Characterization of the receptor-binding domain (RBD) of 2019 novel coronavirus: implication for development of RBD protein as a viral attachment inhibitor and vaccine. |
| 344 | Functional exhaustion of antiviral lymphocytes in COVID-19 patients. |
| 345 | Prudent public health intervention strategies to control the coronavirus disease 2019 transmission in India: A mathematical model-based approach. |
| 346 | Three children who recovered from novel coronavirus 2019 pneumonia. |
| 347 | A COVID-19 Transmission within a family cluster by presymptomatic infectors in China. |
| 348 | Lack of SARS-CoV-2 RNA environmental contamination in a tertiary referral hospital for infectious diseases in Northern Italy. |
| 349 | Spike protein recognition of mammalian ACE2 predicts the host range and an optimized ACE2 for SARS-CoV-2 infection. |
| 350 | High-flow nasal-oxygenation-assisted fibreoptic tracheal intubation in critically ill patients with COVID-19 pneumonia: a prospective randomised controlled trial. |
| 351 | Time Kinetics of Viral Clearance and Resolution of Symptoms in Novel Coronavirus Infection. |
| 352 | Lung Recruitability in SARS-CoV-2 Associated Acute Respiratory Distress Syndrome: A Single-center, Observational Study. |
| 353 | Protein structure and sequence re-analysis of 2019-nCoV genome refutes snakes as its intermediate host or the unique similarity between its spike protein insertions and HIV-1. |
| 354 | SARS-CoV-2 turned positive in a discharged patient with COVID-19 arouses concern regarding the present standard for discharge. |
| 355 | Predicting the angiotensin converting enzyme 2 (ACE2) utilizing capability as the receptor of SARS-CoV-2. |
| 356 | Effect of gastrointestinal symptoms on patients infected with COVID-19. |
| 357 | The characteristics and clinical value of chest CT images of novel coronavirus pneumonia. |
| 358 | Single cell RNA sequencing of 13 human tissues identify cell types and receptors of human coronaviruses. |
| 359 | Estimation of COVID-19 outbreak size in Italy. |
| 360 | Viral dynamics in mild and severe cases of COVID-19. |
| 361 | Prolonged presence of SARS-CoV-2 viral RNA in faecal samples. |
| 362 | Case report of COVID-19 in a kidney transplant recipient: Does immunosuppression alter the clinical presentation? |
| 363 | Clinical Features and Treatment of COVID-19 Patients in Northeast Chongqing. |
| 364 | Prepare to adapt: Blood supply and transfusion support during the first 2 weeks of the 2019 Novel Coronavirus (COVID-19) pandemic affecting Washington State. |
| 365 | A Unique Protease Cleavage Site Predicted in the Spike Protein of the Novel Pneumonia Coronavirus (2019-nCoV) Potentially Related to Viral Transmissibility. |
| 366 | Profiling Early Humoral Response to Diagnose Novel Coronavirus Disease (COVID-19). |
| 367 | Rhabdomyolysis as Potential Late Complication Associated with COVID-19. |
| 368 | The clinical characteristics of pneumonia patients co-infected with 2019 novel coronavirus and influenza virus in Wuhan, China. |
| 369 | Knowledge and Perceptions of COVID-19 Among the General Public in the United States and the United Kingdom: A Cross-sectional Online Survey. |
| 370 | Hydroxychloroquine, a less toxic derivative of chloroquine, is effective in inhibiting SARS-CoV-2 infection in vitro. |
| 371 | Social Capital and Sleep Quality in Individuals Who Self-Isolated for 14 Days During the Coronavirus Disease 2019 (COVID-19) Outbreak in January 2020 in China. |
| 372 | First Pediatric Case of Coronavirus Disease 2019 in Korea. |
| 373 | CT appearance of severe, laboratory-proven coronavirus disease 2019 (COVID-19) in a Caucasian patient in Berlin, Germany. |
| 374 | Clinical features of severe pediatric patients with coronavirus disease 2019 in Wuhan: a single center's observational study. |
| 375 | Co-infection with SARS-CoV-2 and Human Metapneumovirus. |
| 376 | Association of radiologic findings with mortality of patients infected with 2019 novel coronavirus in Wuhan, China. |
| 377 | Initial Investigation of Transmission of COVID-19 Among Crew Members During Quarantine of a Cruise Ship - Yokohama, Japan, February 2020. |
| 378 | Artificial Intelligence Distinguishes COVID-19 from Community Acquired Pneumonia on Chest CT. |
| 379 | Temporal Changes of CT Findings in 90 Patients with COVID-19 Pneumonia: A Longitudinal Study. |
| 380 | Characteristics and Outcomes of 21 Critically Ill Patients With COVID-19 in Washington State. |
| 381 | Serial Interval of COVID-19 among Publicly Reported Confirmed Cases. |
| 382 | A doubt of multiple introduction of SARS-CoV-2 in Italy: a preliminary overview. |
| 383 | A doubt of multiple introduction of SARS-CoV-2 in Italy: a preliminary overview. |
| 384 | The impact of high-flow nasal cannula (HFNC) on coughing distance: implications on its use during the novel coronavirus disease outbreak. |
| 385 | CT image of novel coronavirus pneumonia: a case report. |
| 386 | Analysis of COVID-19 infection spread in Japan based on stochastic transition model. |
| 387 | SARS-CoV-2 enterocolitis with persisting to excrete the virus for about two weeks after recovering from diarrhea: A case report. |
| 388 | Host susceptibility to severe COVID-19 and establishment of a host risk score: findings of 487 cases outside Wuhan. |
| 389 | Insight into COVID-2019 for pediatricians. |
| 390 | A Trial of Lopinavir-Ritonavir in Adults Hospitalized with Severe Covid-19. |
| 391 | SARS-CoV-2 Infection in Children. |
| 392 | Pregnancy and Perinatal Outcomes of Women With Coronavirus Disease (COVID-19) Pneumonia: A Preliminary Analysis. |
| 393 | Skin damage among healthcare workers managing coronavirus disease-2019. |
| 394 | 2019 Novel Coronavirus (COVID-19) Pneumonia: Serial Computed Tomography Findings. |
| 395 | Emerging 2019 Novel Coronavirus (2019-nCoV) Pneumonia. |
| 396 | Ventilatory Ratio in Hypercapnic Mechanically Ventilated Patients with COVID-19 Associated ARDS. |
| 397 | Risk of COVID-19 importation to the Pacific islands through global air travel. |
| 398 | Transmission electron microscopy imaging of SARS-CoV-2. |
