# Supplementary material for: Global academic response to COVID‐19: Cross‐sectional study
Source: Learn Publ. 2020 Jul 1;33(4):385–93. doi: 10.1002/leap.1317 (PMC7362145; doi:10.1002/leap.1317)
Supplement: Supplementary file 2 — Appendix S2 MERS study title. [file LEAP-33-385-s002.docx]

| **ID** | **MERS Study Title** |
| --- | --- |
| 1 | A Fluorogenic Peptide Cleavage Assay to Screen for Proteolytic Activity: Applications for coronavirus spike protein activation. |
| 2 | Elevated human dipeptidyl peptidase 4 expression reduces the susceptibility of hDPP4 transgenic mice to middle east respiratory syndrome coronavirus infection and disease. |
| 3 | Mutations in the spike protein of Middle East respiratory syndrome coronavirus transmitted in Korea increase resistance to antibody-mediated neutralization. |
| 4 | Lysosomal proteases are a determinant of coronavirus tropism. |
| 5 | Enhanced ability of oligomeric nanobodies targeting MERS coronavirus receptor-binding domain. |
| 6 | Interferon regulatory factor 3-mediated signaling limits middle-east respiratory syndrome (MERS) coronavirus propagation in cells from an insectivorous bat. |
| 7 | Safe, high-throughput screening of natural compounds of MERS-CoV entry inhibitors using a pseudovirus expressing MERS-CoV spike protein. |
| 8 | Middle East respiratory syndrome coronavirus infection in non-camelid domestic mammals. |
| 9 | Comparative Serological Study for the Prevalence of Anti-MERS Coronavirus Antibodies in High- and Low-Risk Groups in Qatar. |
| 10 | Towards a solution to MERS: protective human monoclonal antibodies targeting different domains and functions of the MERS-coronavirus spike glycoprotein. |
| 11 | Psychological impact of the 2015 MERS outbreak on hospital workers and quarantined hemodialysis patients. |
| 12 | A screening campaign in sea urchin egg homogenate as a platform for discovering modulators of NAADP-dependent Ca2+ signaling in human cells. |
| 13 | NAADP-dependent Ca2+ signaling regulates Middle East respiratory syndrome-coronavirus pseudovirus translocation through the endolysosomal system. |
| 14 | Prevalence of comorbidities in cases of Middle East respiratory syndrome coronavirus: A retrospective study. |
| 15 | Characterization of the lipidomic profile of human coronavirus-infected cells: Implications for lipid metabolism remodeling upon coronavirus replication. |
| 16 | Proficiency testing for the detection of Middle East respiratory syndrome coronavirus demonstrates global capacity to detect Middle East respiratory syndrome  coronavirus. |
| 17 | Serologic Follow-up of Middle East Respiratory Syndrome Coronavirus Cases and Contacts - Abu Dhabi, United Arab Emirates. |
| 18 | Potent MERS-CoV fusion inhibitory peptides identified from HR2 domain in spike protein of bat coronavirus HKU4. |
| 19 | Complement receptor c5ar1 inhibition reduces pyroptosis in hdpp4-transgenic mice infected with mers-cov. |
| 20 | Combining a fusion inhibitory peptide targeting the mers-cov s2 protein hr1 domain and a neutralizing antibody specific for the s1 protein receptor-binding domain (Rbd)  showed potent synergism against pseudotyped mers-cov with or without mutations in rbd. |
| 21 | Structure and oligomerization state of the C-terminal region of the Middle East respiratory syndrome coronavirus nucleoprotein. |
| 22 | Depression as a mediator of chronic fatigue and post-traumatic stress symptoms in middle east respiratory syndrome survivors. |
| 23 | Epidemiological study of Middle East respiratory syndrome coronavirus infection in dromedary camels in Saudi Arabia, April-May 2015. |
| 24 | Replication of MERS and SARS coronaviruses in bat cells offers insights to their ancestral origins. |
| 25 | Detection of distinct MERS-Coronavirus strains in dromedary camels from Kenya, 2017. |
| 26 | Critically ill healthcare workers with the middle east respiratory syndrome (MERS): A multicenter study. |
| 27 | CD8+ T cells responding to the middle east respiratory syndrome coronavirus nucleocapsid protein delivered by vaccinia virus MVA in mice. |
| 28 | An Atypical Case of Middle East Respiratory Syndrome in a Returning Traveler to Korea from Kuwait, 2018. |
| 29 | Structural and biochemical characterization of endoribonuclease nsp15 encoded by middle east respiratory syndrome coronavirus. |
| 30 | The endonucleolytic RNA cleavage function of nsp1 of middle east respiratory syndrome coronavirus promotes the production of infectious virus particles in specific  human cell lines. |
| 31 | Genetic Evidence of Middle East Respiratory Syndrome Coronavirus (MERS-Cov) and Widespread Seroprevalence among Camels in Kenya. |
| 32 | Airflow as a possible transmission route of middle east respiratory syndrome at an initial outbreak hospital in Korea. |
| 33 | Absence of neutralizing activity in serum 1 year after successful treatment with antivirals and recovery from mers in south korea. |
| 34 | Unexpected Receptor Functional Mimicry Elucidates Activation of Coronavirus Fusion. |
| 35 | Associations Between Hand Hygiene Education and Self-Reported Hand-Washing Behaviors Among Korean Adults During MERS-CoV Outbreak. |
| 36 | Post-exposure prophylaxis for Middle East respiratory syndrome in healthcare workers. |
| 37 | Development and Evaluation of a Multiplexed Immunoassay for Simultaneous Detection of Serum IgG Antibodies to Six Human Coronaviruses. |
| 38 | The Human Sodium Iodide Symporter as a Reporter Gene for Studying Middle East Respiratory Syndrome Coronavirus Pathogenesis. |
| 39 | Arch-shaped multiple-target sensing for rapid diagnosis and identification of emerging infectious pathogens. |
| 40 | Scope and extent of healthcare-associated Middle East respiratory syndrome coronavirus transmission during two contemporaneous outbreaks in Riyadh, Saudi Arabia,  2017. |
| 41 | Structural basis of development of multi-epitope vaccine against middle east respiratory syndrome using in silico approach. |
| 42 | Evaluation of visual triage for screening of Middle East respiratory syndrome coronavirus patients. |
| 43 | Functional analysis of potential cleavage sites in the MERS-coronavirus spike protein. |
| 44 | Agent-based modeling for super-spreading events: A case study of mers-cov transmission dynamics in the republic of korea. |
| 45 | An electrochemical immunosensor for the corona virus associated with the Middle East respiratory syndrome using an array of gold nanoparticle-modified carbon  electrodes. |
| 46 | Acute respiratory infection in human dipeptidyl peptidase 4-transgenic mice infected with Middle East respiratory syndrome coronavirus. |
| 47 | TMPRSS2 contributes to virus spread and immunopathology in the airways of murine models after coronavirus infection. |
| 48 | Efficacy of an adjuvanted middle east respiratory syndrome coronavirus spike protein vaccine in dromedary camels and alpacas. |
| 49 | Evolutionary relationship analysis of Middle East respiratory syndrome coronavirus 4a and 4b protein coding sequences. |
| 50 | Co-localization of Middle East respiratory syndrome coronavirus (MERS-CoV) and dipeptidyl peptidase-4 in the respiratory tract and lymphoid tissues of pigs and llamas. |
| 51 | The perceived effectiveness of MERS-CoV educational programs and knowledge transfer among primary healthcare workers: A cross-sectional survey. |
| 52 | A high-throughput inhibition assay to study MERS-CoV antibody interactions using image cytometry. |
| 53 | Immune Responses to Middle East Respiratory Syndrome Coronavirus during the Acute and Convalescent Phases of Human Infection. |
| 54 | Middle East respiratory syndrome coronavirus intermittent positive cases: Implications for infection control. |
| 55 | Development and validation of different indirect ELISAs for MERS-CoV serological testing. |
